# Fracture resistance of tooth restored with four glass fiber post systems of varying surface geometries-An *in vitro* study

**DOI:** 10.4317/jced.52491

**Published:** 2016-02-01

**Authors:** Adhikesavan Jayasenthil, Emmanuel Solomon-Sathish, Potluri Venkatalakshmi-Aparna, Sunderasan Balagopal

**Affiliations:** 1 MDS, Reader, Department of Conservative Dentistry and Endodontics, Adhiparasakthi Dental College and Hospital, Melmaruvathur, Tamilnadu, India; 2 MDS, Professor, Department of Conservative Dentistry and Endodontics, Rajah Muthiah Dental College and Hospital, Chidambaram; 3 MDS, Reader, Department of Oral Medicine and Radiology, Ragas Dental College and Hospital, Chennai; 4 MDS, Professor and Head, Department of Conservative Dentistry and Endodontics, Tagore Dental College and Hospital, Chennai

## Abstract

**Background:**

The purpose of this study was to relate the fracture resistance of endodontically treated teeth in relation to post geometry.

**Material and Methods:**

Forty single rooted mandibular premolars were instrumented by step - back technique and obturated by lateral condensation. Forty teeth were randomly divided into four groups: Reforpost glass fiber X-ray®, RelyX®, Exacto conical® and Parapost Fiber Lux®. The post spaces were prepared using respective drills and luted. The core build up was done and metal crowns were luted. Fracture resistance was determined in universal testing machine. The statistical analysis was done using one way ANOVA and post hoc Tukey Kramer test.

**Results:**

The teeth restored with Reforpost showed highest fracture resistance followed by Parapost and Exacto conical. The teeth restored with RelyX showed least fracture resistance. The teeth restored with Parapost had less unfavourable fracture followed by exacto conical.

**Conclusions:**

Parallel design had less number of catastrophic failure and had better fracture resistance.

** Key words:**Fracture resistance, glass fiber post, post geometry, stress.

## Introduction

Endodontically treated teeth are compromised by coronal destruction from dental caries, fractures, and previous restorations (1). These compromised teeth should be reconstructed to regain their original form and function. One of the common methods of restoration of such broken down tooth is with the use of intra-radicular posts. It has been a topic of many researches whether the use of post helps in regaining the diminished fracture resistance of the remaining tooth and the various benefits of intra-radicular post (2).

The fracture resistance of the residual tooth is the result of a combination of clinical factors, such as quality and quantity of remaining tooth structure, preparation of remaining tooth, and choice of restorative technique, material and integration of the post. All these factors affect the stress distribution. The basic behavior of fracture is thus determined by stress conditions in combination with strength properties of the individual materials (3,4).

The Glass fiber post has been reported to exhibit high fatigue strength, high tensile strength, and a modulus of elasticity closer to dentin (5). They reportedly reduce the risk of root fracture and have higher survival rates in case of intermittent loading (6).

In prefabricated metal posts it has been proved that passive parallel sided posts have least stress than tapered or threaded posts (7,8). Well adapted tapered posts are reported to causes wedging effect that can fracture the root (9). Texeira ECN (10) has concluded in their study that post geometry plays important role in the mechanical behavior of the posts (10). So this study was done to relate the fracture resistance of endodontically treated teeth in relation to post geometry.

## Material and Methods

The study protocol has been approved Institutional review board Rajah Muthiah Dental College. 40 single rooted mandibular premolars extracted for orthodontic reasons with similar root dimensions were included in this study. The teeth with extremely curved roots, teeth with fracture lines, severely calcified roots and root caries were excluded from the study. The teeth samples were sectioned just below the cement – enamel junction with diamond disc under coolant, such that the remaining root length will be 13 ± 1 mm (11).

-Endodontic Treatment.

The canal patency was checked with 10 K- file (12). The cleaning and shaping of the canals were done using step-back technique using K-files (Mani Inc.), with apical enlargement of 40 size K-file and step-back with 45, 50, 55 with 1mm reduction in working length for each files respectively. The canal was irrigated frequently with 5.25% sodium hypochlorite and 17% EDTA and finally rinsed with 5ml saline.

The canal was dried with paper points. The master cone tug back was confirmed. The obturation was done with Gutta-Percha (Dentsply) and AH plus® (Dentsply) as sealer by lateral condensation technique.

The teeth were stored in saline at room temperature for 24hrs for the sealers to set. The teeth samples were divided into 4 groups.

Group I - Reforpost glass fiber X-ray (Angelus)® Multiple taper post

Group II - RelyX (3M ESPE) ® Tapered post

Group III - Exacto conical (Angelus) ® cylindro conical post

Group IV - Parapost Fiber Lux (Coltene/Whaledent) ® Parallel post

-Post Endodontic Restoration:

The apical diameters of the posts were kept constant as 9mm (7mm inside the canal and 5mm outside the canal). The post spaces were prepared for 7mm using respective drills supplied by the manufacturer.

The canal walls of post spaces were acid etched with 37% ortho phosphoric acid (Total etch®, Ivoclar vivadent). The bonding agent (Adper single bond®, 3M ESPE) was applied and cured as per manufacturer’s instruction. The posts were sectioned to 12mm with diamond bur under coolant. The surface treatments of the posts were done with 10% hydrofluoric acid (Condicionador de Porcelan®, Angelus) and then silanization of posts were done with silane (Silano®, Angelus).

The posts were luted with dual cure resin cement (U100RelyX®, 3M ESPE). The posts were seated with finger pressure for 10 seconds and then excess was wiped off and light cured for 40 seconds using light cure unit. The core build up was done with direct nano hybrid composite (Ceram.X® Dentsply) and light cured. The matrix was adapted over the core using vacuum adaptus system. Using these matrices core build up was done for the rest of the samples.

Wax pattern was fabricated over the cores for full metal crown. Metal crowns were fabricated in the laboratory. The metal crowns were luted using Zinc Phosphate luting cement (Dentsply) (13).

To simulate periodontal ligament first the roots were covered with uniform layer of wax 2mm below cervical margin and immersed in the self cure acrylic resin in the custom fabricated metal mould of size 2.5´2.5´2.5cm.

The resin blocks were de-waxed by immersing them in hot water. The light body impression material (Speedx®, coltene whale-dent) was mixed and coated over the roots and the teeth were repositioned in the resin blocks such that 2mm of the root protruding out of the block, the excess material was removed.

-Fracture resistance test:

To hold the resin at 45 angulations a metal vee block was fabricated. The resin blocks with the teeth were mounted on the metal blocks and subjected to static compressive load at 45° angulations at a crosshead speed of 1mm/minute using universal testing machine (LR100K, Lloyd), until there was a sudden drop of the stress-strain curve. The load to fracture was measured.

The location of failure in all samples was recorded. When the teeth exhibited vertical or oblique fractures extending into or below the surrounding acrylic resin block, the fracture was considered to be unfavorable and non-restorable. Fractures of the tooth above the acrylic resin block were considered restorable and more favorable.

-Statistical analysis:

The statistical analysis was done using one way ANOVA and post hoc Tukey Kramer test was done for intergroup comparison at *p* value < 0.05.

## Results

-Fracture resistance:

Individual fracture resistance values are displayed in  Table 1 and statistics of the fracture strength values (N) of the experimental groups are displayed in  Table 2. The results showed that the fracture resistance of the Group 1 (Reforpost®) was significantly higher than all other groups. The Group 2 (RelyX®) had the least fracture resistance. The Group 4 (Parapost Fiber Lux®) was also statistically significant than the Group 2 & 3. Group 3 (Exacto conical®) was statistically significant than Group 2.

**Table 1:**
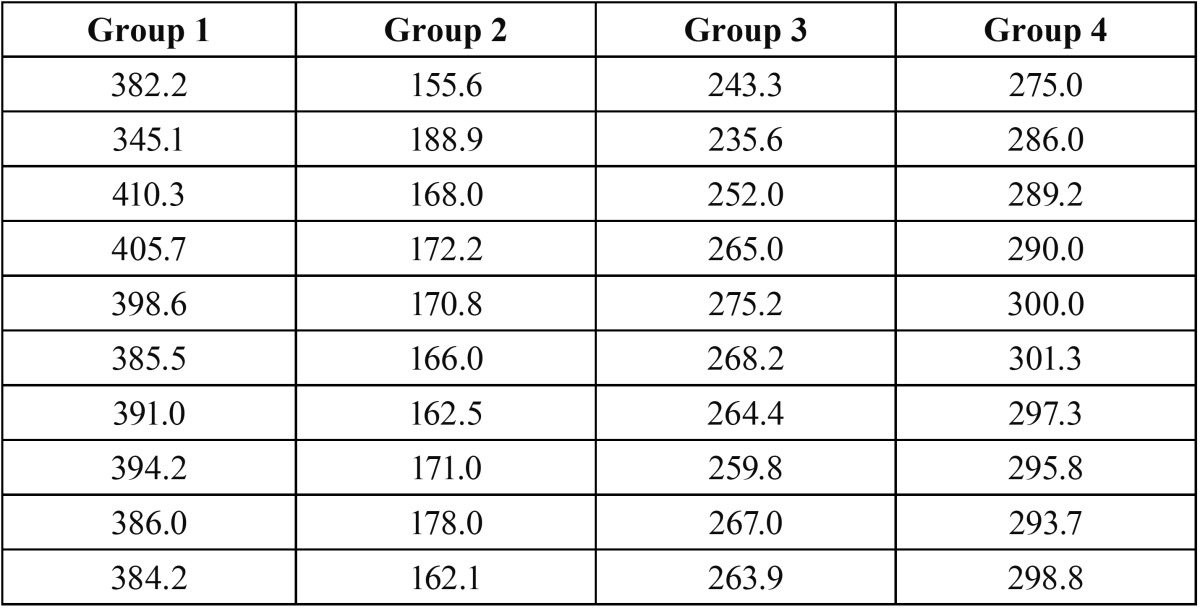
Fracture Resistance of Four Groups in Newtons.

**Table 2:**
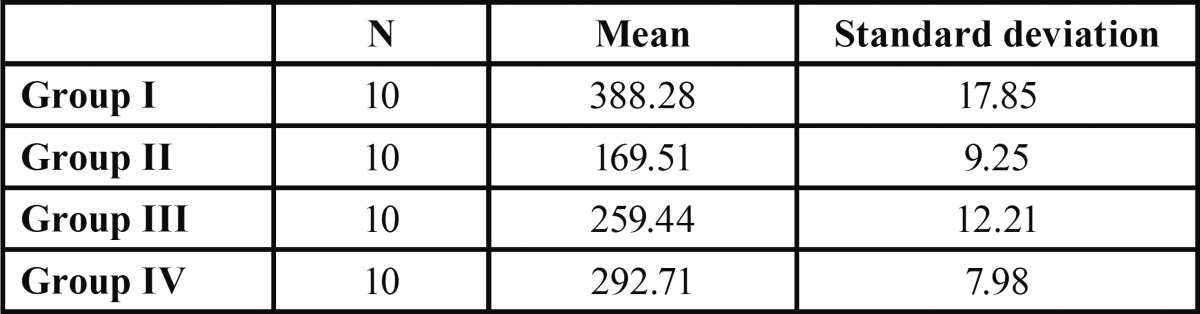
Fracture Resistance of All Groups Mean and Standard Deviation in Newtons.

-Failure modes:

 Table 3 shows the distribution of mode of failure (favourable or unfavourable) of the four experimental groups after the mechanical testing. Group 2 had highest amount of unfavourable root fractures i.e. 5 out of 10, followed by Group 1 which had 4 out of 10 and Group 4 had least amount of unfavourable fractures 1 out of 10 samples. Group 3 had 2 unfavourable fractures.

**Table 3:**
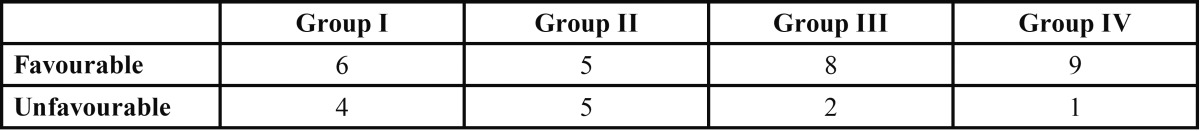
Failure Modes.

As per the obtained statistical results, the p value is significant, *p*<0.001 ( Table 4).

**Table 4:**
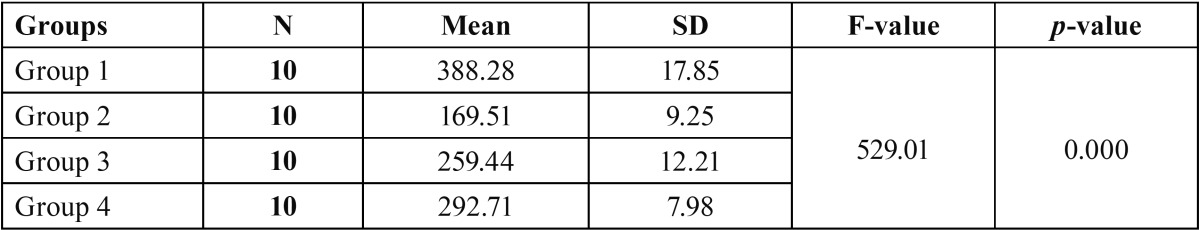
One-way ANOVA results.

## Discussion

Following the root canal therapy the tooth becomes weaker because of loss of the sound tooth structure during cleaning and shaping. Furthermore these teeth often have little coronal tooth tissue to retain a core and final restoration which needs a post (1).

The posts with higher modulus of elasticity cause the root fractures when they fail. Comparatively the low modulus posts like fiber posts causes only little damage to the remaining tooth structure. The prefabricated endodontic posts are classified according to shape and surface configuration. They are basically parallel, tapered or parallel tapered (14).

In one of the *in vitro* studies done to compare the retention, fracture resistance and light transmission behavior of 1 quartz and 3 glass fiber posts. Results indicate parallel posts having better retention as well as fracture resistance. That study was not done in endodontically treated teeth instead inside artificial root canals (10).

The present study was conducted to compare the fracture resistance of teeth restored with four glass fiber posts systems of varying geometry and to correlate the influence of post design in fracture resistance of teeth to glass fiber post systems.

Glass fiber posts were used in this study because these posts have the flexural strength and the modulus of elasticity similar to that of dentin (15,16). In the present study the teeth variation has been standardized with the root sections of all 12 ± 1 mm. The apical size of all the posts have been standardized to 0.9mm. The post length is also standardized to 7mm inside the canal and 5mm outside the canal. The surface conditioning of the posts were done to improve the bond strength of the glass fiber posts to the root canal dentin (17,18). The core build up was standardized by using a custom made polyethylene matrix which had been used in previous studies. The artificial periodontal ligament was used which had been proved by Soares CJ, *et al.* that the root embedment method have a significant effect on fracture resistance of teeth (19).

The results of the present study showed the glass fiber posts with numerous taper showed maximum fracture resistance followed by parallel posts. The highest fracture resistance of the group I could be mainly due to its design. The numerous taper present in the post simulates serrations. Moreover the taper of the post exist only for 3mm from apex and it restarts again. This feature makes this post act almost like a parallel post in the presence of resin cement in the serrations. It had been already reported that parallel posts with serration would have more retention (10).

Next to group I the group IV had better fracture resistance. The higher fracture resistance of the tooth with the parallel post is in conformation with previous studies that also explain the reason as the distribution of stress uniformly along the length of the post (7,20). Another advantage of this group over the other groups was that all other group needed more dentin removal during post space preparation due to their design features.

The least fracture resistance was found with the group II i.e. tapered posts. This is due to the fact that tapered posts concentrate stresses at the shoulder rather than distributing uniformly (21). Moreover the tapered posts provide wedging forces and exhibit wedging effect (7,22).

In case of group III it had two designs combined. It had both parallel design at the coronal end and tapered design at apical end. Because of this design they had better fracture resistance than tapered posts. The parallel portion at the coronal end distributed the stresses uniformly to dentin. The wedging effect is also reduced even though they had a tapered end.

On comparing the failure modes the group IV had only one unfavourable failure. This also was due to the uniform stress distribution of the parallel design. This was consistent with previous studies done on prefabricated metal posts (7,9).

Group I even though, had better fracture resistance than other groups they resulted in more unfavourable failures i.e. 4 samples out of 10. This may be to more removal of dentin during post space preparation. According to Kishen A, *et al.* during post space preparation removal of more amount of inner dentin would result in more catastrophic fractures (23).

The highest amount of unfavourable root fractures occurred in Group 2 (RelyX®) this could have been due to the wedging effect and stress concentration of the tapered design. Furthermore in this group more amount of inner dentin is removed due to the bulk of the post over the coronal end (21,24,25).

Group III had only 2 unfavourable fractures. Though these posts had a tapered end the stress concentration was reduced by the parallel portion. Another reason is that these posts conserved the remaining dentin by its design.

Within the limitations of this in vitro study, it can be concluded that.

• The teeth restored with numerous taper post resulted in higher fracture resistance.

• The teeth restored with parallel post had better fracture resistance than other group.

• The teeth restored with tapered fiber post resulted in least fracture resistance.

• Parallel design had less number of catastrophic failure followed by cylindro-conical

• Almost in all the fiber posts the failure mode was more favourable.

Further research can be done to compare the different type of esthetic posts such as quartz fiber posts, glass fiber posts and zirconia posts.

-Clinical significance: 

Post design that has good fracture resistance and also doesn’t damage the tooth when it fails is preferred over the post that has maximum fracture resistance and damages the tooth when the post fails.
